# Exploring sex differences in cardiac interoceptive accuracy using the phase adjustment task

**DOI:** 10.1111/psyp.14689

**Published:** 2024-09-25

**Authors:** Ria Spooner, Jonathan M. Bird, Nerea Irigoras Izagirre, Rhea Clemente, Elisa Fernandez Fueyo, Gemma Budworth, Dorina Cocirla, Jennifer Todd, Jane Aspell, Mateo Leganes, Dawn Watling, David Plans, Rebecca Brewer, Jennifer Murphy

**Affiliations:** ^1^ Department of Psychology, Royal Holloway University of London London UK; ^2^ Department of Management University of Exeter Exeter UK; ^3^ Department of Biological Sciences, Royal Holloway University of London London UK; ^4^ Department of Anthropology University College London London UK; ^5^ School of Psychology and Sport Science Anglia Ruskin University Cambridge UK; ^6^ Centre for Psychological Medicine Perdana University Kuala Lumpur Malaysia; ^7^ Louvain Experimental Psychopathology Research Group (LEP), Psychological Science Research Institute UCLouvain Louvain‐la‐Neuve Belgium; ^8^ Developmental Psychopathology Department, Psychology School University of Amsterdam Amsterdam Netherlands; ^9^ Department of Psychology University of Surrey Guildford UK

**Keywords:** interoception, interoceptive accuracy, interoceptive awareness, interoceptive sensibility, sex differences

## Abstract

Previous evidence suggests males and females differ with respect to interoception—the processing of internal bodily signals—with males typically outperforming females on tasks of interoceptive accuracy. However, interpretation of existing evidence in the cardiac domain is hindered by the limitations of existing tools. In this investigation, we pooled data from several samples to examine sex differences in cardiac interoceptive accuracy on the phase adjustment task, a new measure that overcomes several limitations of the existing tools. In a sample of 266 individuals, we observed that females outperformed males, indicative of better cardiac interoceptive accuracy, but had lower confidence than males. These results held after controlling for sex differences in demographic, physiological and engagement factors. Importantly, these results were specific to the measure of cardiac interoceptive accuracy. No sex differences were observed for individuals who completed the structurally identical screener task, although a similar pattern of results was observed in relation to confidence. These surprising data suggest the presence of a female advantage for cardiac interoceptive accuracy and potential differences in interoceptive awareness (metacognition). Possible reasons for mixed results in the literature, as well as implications for theory and future research, are discussed.

## Abstract 2

This paper reports on novel work exploring sex differences in cardiac interoceptive accuracy using a novel test that overcomes limitations of existing measures. At odds with existing evidence, we observed a female advantage for cardiac interoceptive accuracy. This works suggests a need to re‐examine evidence using traditional measures of interoception.

## 1 INTRODUCTION

Interoception—the processing of internal bodily signals—has been associated with a plethora of health conditions and higher order cognitive abilities (Brewer et al., 2021). Given the potential widespread influence of interoception on behavior and well‐being, much research has focused on identifying factors that may contribute to individual differences. One such factor is sex,^1^ where sex differences have been reported both for engagement by interoceptive signals, with females paying more attention (e.g., Franzoi et al., 1989; Grabauskaitė et al., 2017), and the accuracy with which individuals can perceive interoceptive signals, with males outperforming females. These differences in interoceptive accuracy have been reported across a range of signals, including cardiac, respiratory, gastric, blood pressure, blood glucose, nicotine sensitivity, and sexual concordance (Cox et al., 1985; Harver et al., 1993; Pennebaker & Watson, 1988; Perkins, 1999; Prentice & Murphy, 2022; Suschinsky & Lalumière, 2012; Whitehead & Drescher, 1980), though differences in accuracy are typically only observed in laboratory settings (Pennebaker & Roberts, 1992).

The identification of sex differences in interoception has prompted much theoretical work considering the potential cause(s). Such theories include differences in signal strength, socialization, stress exposure, the use of external cues, brain differences, and physical change across development (e.g., menstruation, pregnancy, menopause), among others (Ma‐Kellams et al., in press; Harshaw, 2015; Longarzo et al., 2021; Murphy et al., 2019; Pennebaker & Roberts, 1992; Prentice et al., 2022). However, while extensive meta‐analytic work supports the presence of sex differences in interoceptive accuracy—specifically sex differences in cardiac interoceptive accuracy (Prentice & Murphy, 2022)—where males have been shown to outperform females on both heartbeat counting and heartbeat detection measures (Prentice & Murphy, 2022), concerns have been raised about the validity of such measures. Specifically it has been argued that these measures may be contaminated by estimation strategies resulting in false positives, and do not account for individual differences in the timing at which individuals perceive an external stimulus to be synchronous with their heartbeat resulting in false negatives, respectively (Brener & Ring, 2016; Desmedt et al., 2023). Accordingly, it is necessary to examine sex differences in cardiac interoceptive accuracy using newly developed tasks that overcome these weaknesses.

In the present study, we pooled together all available data using the recently developed Phase Adjustment Task (PAT; Plans et al., 2021). In the PAT, participants are presented with a series of tones that are triggered by their heartbeat, but are out of phase with their heartbeat. They are asked to adjust a virtual dial to align the tones with their heartbeats. As the starting phase is random across trials, the consistency of the participant's selected delays is taken to represent their accuracy. This task overcomes the limitations of existing tasks of cardiac interoceptive accuracy that may be influenced by estimation strategies, as knowledge of heart rate is of no practical use when completing the task, as participants are always presented with tones at their heart rate. The task also accounts for individual differences in delay preferences, as participants can select any delay and accuracy is inferred from the consistency of responses. These attributes of the task mean that it has been described as one of the most promising new tools for assessing cardiac interoceptive accuracy (Desmedt et al., 2023). While we expected that males would outperform females, in alignment with previous research (Prentice & Murphy, 2022), we made no specific prediction given the limitations of existing tasks and evidence suggesting that diverse results may be obtained when different measures of cardiac interoceptive accuracy are used (Todd et al., 2024).

## 2 METHODS

### 2.1 Ethics

Three of the four datasets used in the present investigation were derived from studies that received ethical approval from the Royal Holloway Ethics Subcommittee following their reviewing procedures. The fourth dataset was derived from a study that received ethical approval by the Rutgers University Institutional Review Board. All participants provided informed consent and were fully debriefed after task completion.

### 2.2 Participants

Initially, we sought to pool data from every study that involved the PAT to date that had recorded sex assigned at birth (see Table 1 for wording across studies). While most studies included an option for participants to indicate “Other” alongside “Male” and “Female”, no participant selected this option. We excluded studies that recruited individuals from one sex only or a specific group (e.g., pregnant females), or that involved other tasks of interoception that may introduce interference effects. Furthermore, we excluded studies where demographic data were unavailable at the time data were pooled. After exclusions, four unpublished datasets remained. Datasets 1 and 3 were collected in a laboratory under supervised conditions and Datasets 2 and 4 were completed remotely. These datasets were combined given evidence that PAT scores are comparable when administered in a laboratory and remotely (Spooner et al., under review). Where multiple completions were present (e.g. owing to technical issues), we used the first available completion provided 17 valid trials were available. A cut‐off of 17 valid trials was used given that this was the pre‐registered threshold for Dataset 1 and 15–20 trials provides a reasonable trade‐off between task length and accuracy (https://osf.io/j4dtr; Plans et al., 2021; Todd et al., 2024).

**TABLE 1:** Inclusion and exclusion criteria for each dataset.

	Dataset 1	Dataset 2	Dataset 3	Dataset 4
Recruited *N*	100	143	73	42
Final *N*	86	116	54	10
Proportion of sample retained	86%	81.1%	74%	23.8%^a^
*N*_females_	53	63	41	6
Age (*M* and *SD*)	23.09 (7.71) years	31.01 (9.36) years	26.78 (12.13) years	23.50 (3.31) years
Data collection method	Laboratory	Remote	Laboratory	Remote
Inclusion criteria	Aged 18–60 years. Normal/corrected hearing/vision	Aged 18–60 years. Access to an Apple iPhone meeting eligibility requirements. Normal/corrected hearing/vision	Aged 18–65 years. Normal/corrected hearing/vision	Aged 18–35 years; at least one night of heavy drinking in the past 30 days (4 or 5 alcoholic drinks in less than 2 h for females and males, respectively); consume more than 5 drinks per week; one hangover in past 30 days; one blackout episode in the past 6 months. No self‐reported history of cardiovascular or mental health disorder. Access to an Apple iPhone meeting eligibility requirements
Control Task	Yes	Yes	No	No
Mental health (% reporting)	16.3%	0.9% (9.5% declined or missing)	0%	Not recorded
Physical health (% reporting)	12.8%	Not recorded	Not recorded	Not recorded
Sex question	What is your sex assigned at birth? (Male/Female/Other)	Indicate the sex/please select the sex you were assigned at birth (Male/Female/Other)	Indicate the sex you were assigned at birth (Male/Female/Other)	What sex were you assigned at birth, on your original birth certificate? (Male/Female)

^a^ Note that data loss was higher for Dataset 4 owing to some technical issues that resulted in overwriting of the baseline data due to the longitudinal nature of the project.

We excluded participants for whom demographic data were missing (*N* = 3 from Dataset 2, *N* = 2 from Dataset 3) and one participant who took part after we began data processing (Dataset 2). We excluded one participant from Dataset 3, as their self‐reported age was beyond the inclusion criteria for the associated study (see Table 1). Finally, we excluded three participants who, due to a technical problem with the application, completed additional trials. The final sample comprised 266 participants (*M*_age_ = 27.31 years, *SD*_age_ = 9.96 years, *N*_females_ = 163). This sample size provides >95% power to detect a medium effect size (Cohen's *d* = 0.50), two‐tailed. Please see Table 1 for full inclusion criteria for each study and the associated sample sizes. Note that PAT consistency scores did not vary across the four datasets as assessed by a one‐way analysis of variance (*F*(3, 262) = 0.596, *p* = .618), nor classifications at any of the three Bayes Factor Levels when assessed by chi‐squared analysis (*p*s > .250), justifying our decision to pool these samples.

### 2.3 Materials

#### 2.3.1 Phase adjustment task

All participants completed the PAT described in Plans et al. (2021), implemented using a purpose‐built smartphone application. In this task, participants are required to hold their finger over the smartphone camera and flash, and heartbeats are recorded via photoplethysmography. After a 2‐min baseline recording period to capture the heart rate, participants were presented with task instructions (see Supporting information). In the PAT task, participants are presented with a series of tones that are triggered by their heartbeat, but are out of phase with their heartbeat. They are required to adjust a virtual dial to advance or delay the tones until they perceive them to be synchronous. After confirming their response, participants are required to rate their confidence in having successfully completed the previous trial on a 10‐point scale (0 = “Not at all confident”, 9 = “Extremely confident”) and then automatically advance to the next trial. Every five trials, participants are also asked to indicate the location from which they felt their heartbeat using an on‐screen body map. Participants completed two practice trials, followed by 20 main task trials. It is noteworthy that tones were triggered by heartbeats. This differs from the original Plans et al. (2021) investigation where an algorithm was used to predict the occurrence of the next heartbeat from the preceding 3 s. This change from using predicted to actual heartbeats was implemented due to the possibility that the accuracy of the algorithms predictions may vary slightly across participants as a function of heart rate variability (HRV). Using heartbeats to trigger tones removes the possibility of differences in the accuracy of predictions.

#### 2.3.2 Screener task

Participants in Dataset 1 and most participants in Dataset 2 completed a screener task prior to the PAT. The screener task was identical to the PAT, except that participants were presented with two tones on each trial, one triggered by their heartbeat and the other triggered by the heartbeat but out of phase, and were required to adjust an on‐screen dial until they perceived the two tones to be synchronous (see Plans et al., 2021; Study 2). Participants heartbeat was recorded throughout the task, but they were not explicitly informed that one tone was triggered by their heartbeat. As the PAT involves matching a tone to one's heartbeat, the purpose of the screener task was to ensure that participants were able to match two stimuli. Ensuring participants pass the screener thus allows us to conclude that differences in PAT performance are not likely due to an inability to match stimuli, or wider cognitive impairment etc. Participants completed two practice trials, followed by 20 main task trials.

### 2.4 Procedure

Testing procedures varied slightly across the participant groups in each dataset. For Dataset 1, participants completed the screener task and the PAT on the same day, separated by ~20 min of questionnaires. Each participant was supervised by either RS, RC or NII and they completed the tasks on Apple iPhones provided by the researchers. For Dataset 2, participants were recruited via Testable. Data were pooled from two projects for the purpose of this study to make use of all available data collected by JM and EFF. For both projects, participants were initially screened using either Gorilla or Qualtrics to ensure their smartphones met the application requirements. A small number of participants completed questionnaires and a behavioral task on Gorilla prior to completing the applications. After demonstrating eligibility, participants were presented with instructions for downloading the applications. The majority of participants were then only invited to complete the PAT application at a later time if they had completed the screener application to a sufficient standard (scores >0.42^2^), though a small minority of online participants did not complete the screener as it was not included in the first run of participants. Of the 96 participants in Dataset 2 that completed the screener, the average time between completion of the screener and PAT was 45 days, but this varied considerably across participants (*M* = 44.91 days, *SD* = 101.80 days, Range: 0–498 days). For Dataset 3, participants took part in a laboratory study supervised by GB, DC or other trained research assistants and completed the PAT on Apple iPhones provided by the researchers. Prior to completion of the PAT, participants completed questionnaires and a behavioral task on Gorilla. For Dataset 4, data were collected in the context of a study examining the effects of hangovers on interoception using ecological momentary assessment. Participants were recruited via word of mouth, online fora (i.e., reddit), student lists, and through flyers pasted in bars and breweries in Continental USA. For Dataset 4, we only included data from the initial baseline session where participants were told to refrain from drinking alcohol for 24 h.

### 2.5 Analysis strategy

PAT data were initially exported in JSON format. We imported this data into R Studio (v2023.12.1) and used the tidyr package to render the data tidy (i.e., each row corresponding to a PAT trial). We extracted the beat‐to‐beat (RR) intervals from the 2 min baseline heart rate data. Thereafter, the RHRV R package was employed to calculate several time domain HRV metrics (i.e., RMSSD, pNN50, SDNN). Engagement metrics were also computed for each PAT trial, such as the number of unique dial positions and the time taken to completion. After removing each participant's practice trials (*n* = 2), we checked several quality filters, so that trials with 0 delays or ≤4 heart rate values were removed, owing to a lack of user engagement. We excluded participants who had registered <17 valid trials (out of 20). To ensure that participants had equal trial numbers, we selected each participant's first 17 trials (while discarding additional trials) to allow for the computation of aggregate engagement metrics (e.g., total time spent on trials; mean time spent on trials; mean number of dial turns; number of valid trials) and consistency scores. Example code can be found at https://github.com/huma‐engineering/Phase‐Adjustment‐Task.

Consistency scores refer to the consistency of the selected delays across PAT trials and are employed to help determine whether an individual is interoceptive or non‐interoceptive. Briefly, consistency scores are computed from measures of angular similarity representing the phase relationship between heartbeats and tones on a trial‐by‐trial basis. If angles are close to one another, the corresponding score is close to 1. Conversely, if angles are randomly positioned around the dial, the corresponding consistency score is close to 0 (see Plans et al. 2021 for additional details).

The R package AdaptGauss was used to apply a gaussian mixture model to the consistency scores to classify participants as either interoceptive or non‐interoceptive. The mixture model returned two distributions, one for interoceptive and one for non‐interoceptive participants by means of an expectation‐maximization algorithm (Plans et al., 2021). *Z*‐scores were computed for each participant for interoceptive and non‐interoceptive distributions separately. Thereafter, the *z*‐scores were used to calculate the probability of each participant belonging to the interoceptive and noninteroceptive distributions, in alignment with previous PAT‐related research (e.g., Plans et al., 2021; Spooner et al., under review; Todd et al., 2024). Bayes factors (BFs) were calculated as the ratio of the probability of belonging to one of the two distributions over the probability of belonging to the other distribution. Thresholds were used on the BFs, so that participants could be classified as interoceptive, non‐interoceptive, or unclassified (i.e., where there is insufficient evidence for a classification). BFs >3 provided moderate evidence that a participant was interoceptive or non‐interoceptive, BFs > 10 provided strong evidence, and BFs >30 provided very strong evidence (Plans et al., 2021; Todd et al., 2024).

Data were then imported into SPSS for formal analyses and can be accessed at https://osf.io/br9tc/. Formal analyses compared mean consistency scores between male and female participants using independent samples *t*‐tests and classification scores using chi‐squared analyses. Normality assumptions were checked using visual inspection, and where deviations from normality were observed (for engagement metrics and heart rate metrics), non‐parametric equivalents were used. One outlier was present for consistency scores, but was retained as excluding this participant did not alter the pattern of significance.^3^ All reported *p*‐values are two‐tailed.

## 3 RESULTS

### 3.1 Full sample

**FIGURE 1:**
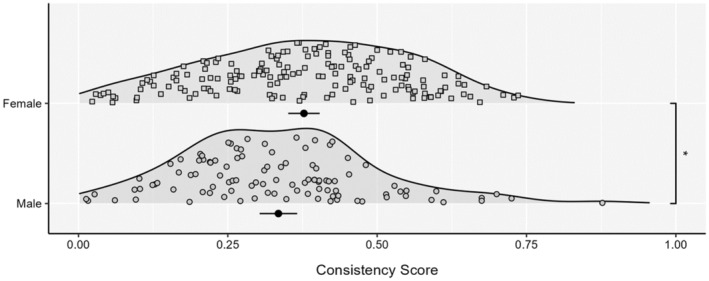
Consistency scores between sexes for the full sample. Each density is accompanied by the mean and 95% CI. *p < .05.

Contrary to predictions, consistency scores were higher in females (*M* = 0.38, *SD* = 0.17) than males (*M* = 0.33, *SD* = 0.16; *t*(264) = 2.04, *p* = 0.042; see Figure 1). Examination of the Bayes Factor Classifications revealed no difference at Bayes Factors 3 or 10, but a significant difference at Bayes Factor 30, with females more likely to be interoceptive than males (see Table 2a). Despite lower objective accuracy, confidence ratings were higher in males (*M* = 5.5, *SD* = 1.92) than females (*M* = 4.6, *SD* = 2.13; *t*(264) = 3.5, *p* < .001).

**TABLE 2:** Bayes factor classifications for the (a) full unscreened sample, (b) unscreened sample matched for engagement and demographics, (c) screened sample, and (d) screened sample matched for engagement and demographics.

Dataset	Males			Females			*χ* ^2^	*p*
	Non‐interoceptive	Unclassified	Interoceptive	Non‐interoceptive	Unclassified	Interoceptive		
(a)								
BF3	30	24	49	39	28	96	3.353	.187
BF10	13	49	41	21	62	80	2.572	.276
BF30	0	76	27	0	94	69	7.109	**.008**
(b)								
BF3	24	22	37	31	21	79	5.638	.060
BF10	9	45	29	14	52	65	4.857	.088
BF30	0	65	18	0	74	57	10.631	**.001**
(c)								
BF3	23	19	32	20	16	59	5.960	.051
BF10	8	39	27	12	32	51	6.364	**.042**
BF30	0	55	19	0	51	44	7.579	**.006**
(d)								
BF3	23	19	32	16	15	47	4.473	.107
BF10	8	39	27	9	29	40	3.949	.139
BF30	0	55	19	0	44	34	5.366	**.021**

*Note*: Significant values are indicated by bold text.

Although we took the a priori decision to match groups where significant differences were observed on physiological or engagement metrics, we made no specific predictions regarding which metrics would differ between males and females. Examination of engagement metrics, heart rate dynamics and further demographic data revealed that males and females significantly differed (or showed a trend for differences) regarding the total number of valid trials available,^4^ resting heart rate, HRV pNN50, and age (see Table 3 for full results). To ensure that sex differences in interoceptive accuracy were not driven by these differences, we took the following approach to match groups. To match groups for age and engagement, we set a threshold of 18 valid trials and excluded all females aged 18 years so that there was no notable difference in engagement between males and females after exclusions (*p*s > .05; *N* = 214; *N*_females_ = 131). In this sample, consistency scores remained higher in females (*M* = 0.39, *SD* = 0.17) than males (*M* = 0.33, *SD* = 0.15; *t*(212) = 2.42, *p* = 0.016). As before, significant differences in classification were only observed at Bayes Factor 30 (see Table 2b). Average confidence ratings remained higher in males (*M* = 5.43, *SD* = 1.92) compared to females (*M* = 4.77, *SD* = 2.23) in this restricted sample (*t*(212) = 2.21, *p* = .028).

**TABLE 3:** Demographic variables, engagement metrics and heart rate data for males and females in both samples.

	Unscreened sample				Screened sample			
	Males	Females	*Z* ^a^	*p*	Males	Females	*Z*	*p*
	*M* (*SD*)	*M* (*SD*)			*M* (*SD*)	*M* (*SD*)		
Total time on task (s)	367.81 (173.45)	396.84 (218.16)	0.633	.507	378.13 (176.02)	401.52 (229.00)	0.165	.869
Mean time per trial (s)	21.64 (10.20)	23.34 (12.83)	0.633	.507	22.24 (10.35)	23.62 (13.47)	0.165	.869
Mean engagement (dial turns) per trial	26.03 (12.72)	29.00 (16.22)	1.36	.174	26.21 (12.68)	29.09 (16.71)	0.894	.372
Number of valid trials	18.80 (1.19)	19.12 (0.96)	1.941	.052	18.93 (1.19)	19.26 (0.87)	1.391	.164
Resting heart rate (bpm)	76.30 (9.79)	79.23 (11.5)	2.57	**.010**	74.51 (9.60)	78.95 (11.52)	3.096	**.002**
Heart rate variability (SDNN)	172.51 (93.17)	172.38 (84.18)	0.210	.883	159.22 (91.56)	168.37 (88.48)	0.821	.412
Heart rate variability (RMSSD)	135.99 (69.60)	140.89 (63.92)	0.763	.445	127.92 (70.72)	139.17 (66.54)	1.191	.233
Heart rate variability (pNN50)	47.42 (20.25)	53.01 (17.73)	2.352	**.019**	45.60 (20.63)	52.75 (18.04)	2.351	**.019**
Age	29.14 (9.86)	26.15 (9.88)	3.490	**<.001**	29.04 (8.95)	26.28 (9.26)	2.678	**.007**

	*N*	*N*	*χ* ^2^	*p*	*N*	*N*	*χ* ^2^	*p*
Mental health (No conditions)^b^	4(91)	11(139)	0.987	.321	4(68)	8(81)	0.410	.680
Physical health (No conditions)^b^	6(27)	5(48)	1.395	.238	6(24)	3(40)	2.773	.096

^a^ As the majority of data were not normally distributed, nonparametric tests were used for all comparisons of engagement and heart rate metrics.

^b^ Note that mental and physical health comparisons make use of available data. These data were not collected for all datasets.

Bold text indicates *p* values <.05.

As significant differences in resting HR and HRV pNN50 remained after the aforementioned matching (*p*s < .05), these variables were subsequently regressed out from the consistency scores. Examination of sex differences in these residuals revealed higher scores in females compared to males (*t*(212) = 2.10, *p* = .037). Finally, as there was still a trend for age differences (*p* = .067) we also regressed this variable from consistency scores alongside HR and HRV pNN50. Again, examination of these residuals still revealed higher consistency scores in females compared to males (*t*(212) = 2.02, *p* = .044).

### 3.2 Screened sample

**FIGURE 2:**
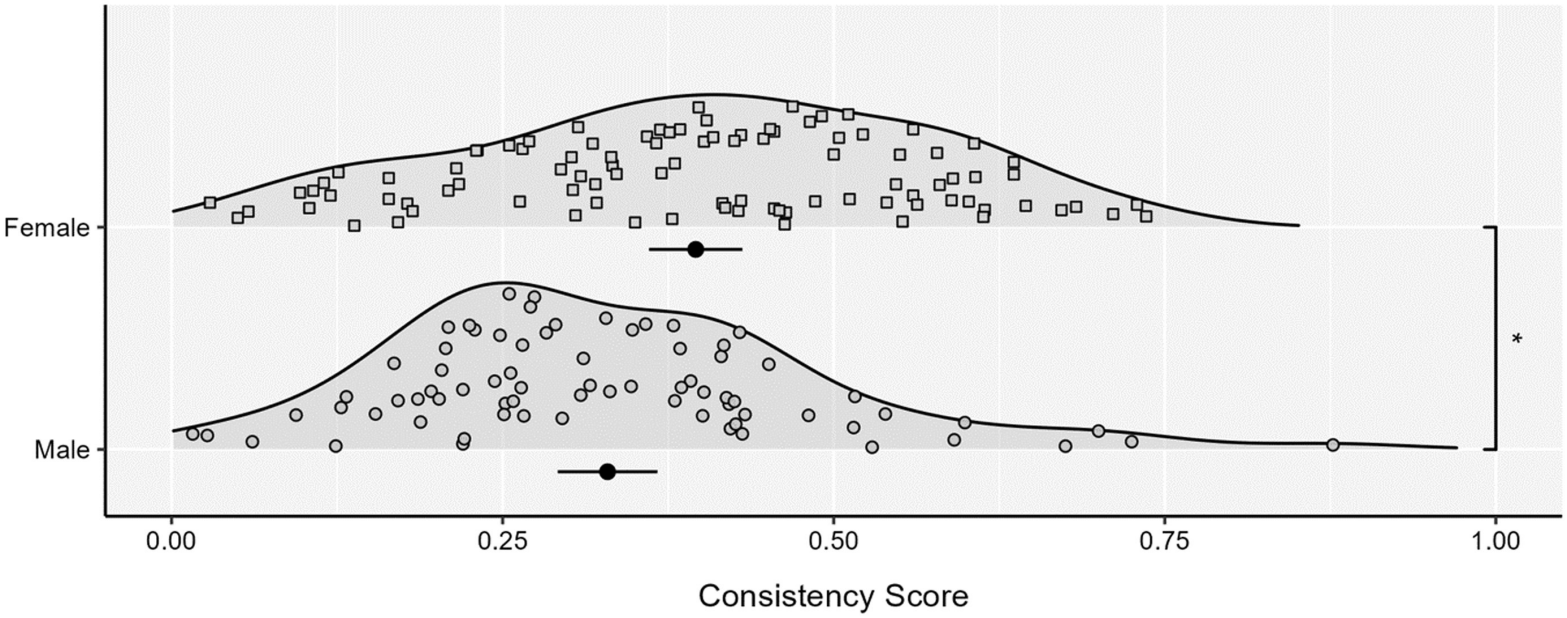
Consistency scores between sexes for the screened sample. Each density is accompanied by the mean and 95% CI. *p < .05.

We repeated the aforementioned analyses restricting the sample to individuals who had completed the screener task and passed with a score >0.42.^5^ This included 169 individuals (*N*_females_ = 95). In the screened sample, consistency scores were higher in females (*M* = 0.40, *SD* = 0.17) than males (*M* = 0.33, *SD* = 0.16; *t*(167) = 2.55, *p* = 0.012; see Figure 2), and this was evident at all three Bayes Factor levels, with significant differences at Bayes Factors 10 and 30 and a trend at Bayes Factor 3 (see Table 2c). Confidence ratings in this sample remained higher in males (*M* = 5.56, *SD* = 1.92) than females (*M* = 4.48, *SD* = 2.10; *t*(167) = 3.45, *p* < .001). No significant difference in screener performance^6^ was observed between males (*M* = 0.90, *SD* = 0.13) and females (*M* = 0.88, *SD* = 0.13; *Z* = 1.507, *p* = .132), but screener confidence was also higher in males (*M* = 7.11, *SD* = 1.28) than females (*M* = 6.36, *SD* = 1.18; *t*(167) = 3.95, *p* < .001). No differences in engagement metrics were found for the screener between males and females or for the PAT (*p*s > .05), but differences in HR, HRV pNN50 and HRV RMSSD were observed (*p*s < .05). Controlling for factors where differences were observed did not change the aforementioned nonsignificant difference between males and females on the screener task. Notably, screener performance was unrelated to performance on the PAT (Spearman's rho(167) = 0.05, *p* = .520).

In this reduced sample, we adopted the same matching approach because similar factors showed significant differences (see Table 3). After matching for age (*N* = 152; *N*_females_ = 78), females (*M* = 0.40, *SD* = 0.17) showed higher consistency scores than males (*M* = 0.33, *SD* = 0.16; *t*(150) = 2.42, *p* = .017). This was reflected at Bayes Factor 30, but not 3 or 10 (see Table 2d). As before, confidence ratings remained higher in males (*M* = 5.56, *SD* = 1.92) than females (*M* = 4.64, *SD* = 2.20; *t*(150) = 2.76, *p* = .007). After controlling for resting HR and HRV pNN50 that differed between males and females (*p*s < .05), consistency scores remained higher for females compared to males (*t*(150) = 2.22, *p* = .028). It is noteworthy that a regression‐based approach (rather than group matching), controlling for age, HRV pNN50 and resting HR still demonstrated significant sex differences, with females outperforming males (*t*(167) = 2.06, *p* = .041).

## 4 DISCUSSION

The aim of the present investigation was to examine whether males and females differed in performance on the PAT, a measure of cardiac interoceptive accuracy that overcomes limitations of previously developed measures. In contrast to existing meta‐analytic work exploring sex differences on the heartbeat counting and heartbeat detection tasks (Prentice & Murphy, 2022), which has found better cardiac interoceptive accuracy in males, we observed significantly better performance in females compared to males. This result held after matching groups on engagement data and physiological factors (i.e., HR and HRV that often vary between males and females; Koenig & Thayer, 2016), as well as restricting inclusion to individuals who had completed the screener task where participants were required to match two tones. Notably, in the screened samples no sex difference was observed for screener performance, suggesting that this sex difference was specific to interoceptive accuracy. In contrast, we observed that males exhibited greater confidence than females, though this result was not specific to interoception, as a similar difference was observed for the screener task.

The finding that females outperformed males on the PAT is at odds with existing evidence showing the opposite pattern of results in the cardiac domain (Prentice & Murphy, 2022), as well as other domains of interoception (see Ma‐Kellams et al., in press for a review). While it is possible that males outperform females in other interoceptive domains—as interoceptive accuracy is not a unitary ability and performance dissociates across domains (Ferentzi et al., 2018; Garfinkel et al., 2016; Steptoe & Vögele, 1992)—one would expect a similar pattern across the cardiac domain. Although there are several possible reasons for this result, it is notable that existing cardiac interoceptive accuracy measures show little correspondence with each other (Hickman et al., 2020), and the limitations of the most frequently administered measures have been well‐described (Brener & Ring, 2016; Desmedt et al., 2023). It is possible that these limitations have contributed to the previously reported male advantage on heartbeat counting and detection tasks. For example, in terms of heartbeat counting, good performance can be achieved via guessing or estimation strategies (Desmedt et al., 2020, 2023; Murphy, Brewer, et al., 2018; Murphy, Millgate, et al., 2018; Phillips et al., 1999; Ring et al., 2015; Ring & Brener, 1996; Windmann et al., 1999). This could explain sex differences, as males may be more likely to recruit estimation strategies than females or have better knowledge of heart rate. Regarding heartbeat detection, one possibility is that males and females differ in the delay at which they perceive an external stimulus to be synchronous with their heartbeat, and that the delays typically used for heartbeat detection tasks are biased towards males (potentially due to differences in pulse transit time and/or the location from which heartbeats are perceived due to differences in body fat; Rouse et al., 1988). However, previous work has not observed sex differences in the temporal locations of heartbeats, albeit in small samples (Ring & Brener, 1992). As studies using the heartbeat detection task vary in the delays used for synchronous and asynchronous intervals (with some using a proportion of the inter‐beat‐interval and some using fixed delays), as well as the instructions used (Brener & Ring, 2016; Hickman et al., 2020), it remains a question for future research whether the previously observed male advantage in heartbeat detection is driven by certain methodological choices. While we did not observe differences between males and females in the amount of time spent on trials, it is also possible that control over the amount of exposure to heartbeats in the PAT plays a role. Heartbeat detection tasks typically restrict exposure to ~10 heartbeats, while in the PAT participants can sample heartbeats for as long as desired. Time spent on trials does not enable us to differentiate all possible effects that control over the amount of exposure to heartbeats may result in. However, it could be that this change reduces pressure on participants, allowing them to sample heartbeats for longer on trials where they have difficulties perceiving heartbeats, which contributes to better performance in females but no overall difference in task time. Future research examining sex differences in cardiac interoceptive accuracy across tasks would be useful for uncovering the reasons for these inconclusive results.

Close inspection of classification scores produced a similar pattern of results, but it should be acknowledged that classification scores only consistently revealed a significant sex difference at the highest Bayes Factor level. As these classifications are more conservative, evidence of consistent sex differences at the most stringent level is reassuring. Surprisingly, however, we observed the opposite pattern of results when examining confidence ratings, wherein males displayed greater confidence than females. Although not specific to interoceptive accuracy, as the same difference was also observed when considering confidence in screener performance, these data suggest that males and females may differ with respect to interoceptive awareness (e.g., metacognitive insight into one's own performance typically assessed by the relationship between trial‐by‐trial confidence ratings and accuracy; Garfinkel et al., 2015) as confidence is higher in males, but accuracy is higher in females. Although results at the group level should be interpreted with caution, it may be that females on average believe their performance to be worse than in reality and males believe the opposite. This could result in sex differences in interoceptive awareness and the propensity to use internal signals (Murphy, 2022), whereby females are less likely to use internal signals (as they believe their perception to be poor when it is in fact good), and males are more likely to use internal signals (as they believe their perception to be good when it is in fact poor). Given evidence of sex differences in the propensity to use internal signals for emotion (Pennebaker & Roberts, 1992; Prentice et al., 2022), further research exploring sex differences in interoceptive awareness and emotion (as well as other factors linked to interoception; see Brewer et al., 2021; Thompson & Voyer, 2014) across domains would be useful for examining the impact of observed sex differences. Although the PAT as presently implemented cannot provide a measure of interoceptive awareness as there is no “correct” answer, and previously used cardiac interoceptive awareness measures (e.g., Garfinkel et al., 2015) are limited as they may not adequately assess accuracy or include too few trials, exploring sex differences in interoceptive awareness using other measures (see e.g., Harrison et al., 2021 for a measure of respiratory interoceptive awareness) will be useful for better understanding patterns observed at the group level.

As well as understanding interoceptive differences between males and females, it would also be beneficial for future research to examine the cause(s) of differences. As detailed in the introduction, various explanations have been proposed, including physical change (e.g., menstruation, pregnancy, menopause), physiological differences (e.g., arousal, brain differences), as well as socialization and stress exposure (e.g., Harshaw, 2015; Longarzo et al., 2021; Ma‐Kellams et al., in press; Murphy et al., 2019; Pennebaker & Roberts, 1992; Prentice et al., 2022). These data cannot speak to the cause(s) of sex differences. Nonetheless, further research examining the contribution of these potential factors is warranted. Such work may also benefit from examining differences across gender variant samples which may help to disentangle the effects of biological and social factors on cardiac interoceptive accuracy.

Despite the utility of these data, limitations must be acknowledged. First, as data pertaining to mental and physical health were not available for all participants, with these factors linked to both sex and interoception, we cannot rule out a contribution of mental and physical health to the pattern of results obtained (Brewer et al., 2021; Kuehner, 2017). However, as we observed no sex differences in mental/physical health where data were available, it is unlikely that differences would be present due to chance. Second, although we controlled for various physiological parameters, not all physiological parameters linked to cardiac interoception were controlled for (e.g., blood pressure and body fat; O'Brien et al., 1998; Rouse et al., 1988). Although sex differences have been reported on the heartbeat counting task even after controlling for blood pressure and body mass index (Murphy, Brewer, et al., 2018), it is still possible that differences in stimulus strength and the use of exteroceptive strategies may play a role. It is noteworthy, however, that this is likely to be the case for all cardiac interoceptive accuracy tasks where sex differences have been explored (Desmedt et al., 2023). Moreover, as previous evidence suggests that higher blood pressure and lower body fat are typically linked to better cardiac interoceptive accuracy (O'Brien et al., 1998; Rouse et al., 1988), and these factors are typically higher and lower respectively in males compared to females, this is also unlikely to explain the sex difference observed here.

In conclusion, we found evidence of sex differences in cardiac interoceptive accuracy using the PAT, whereby females outperformed males, but males were more confident than females. These findings stand in opposition to the extant literature. However, they provide novel theoretical avenues for exploring sex differences in the relationship between interoception, health and cognition, and suggest a need to re‐examine results in the literature using novel tests that overcome the limitations of existing tools.

## AUTHOR CONTRIBUTIONS

**Ria Spooner:** Data curation; formal analysis; methodology; project administration; writing – original draft; writing – review and editing. **Jonathan M. Bird:** Data curation; formal analysis; visualization; writing – original draft; writing – review and editing. **Nerea Irigoras Izagirre:** Data curation; project administration; writing – review and editing. **Rhea Clemente:** Data curation; project administration; writing – review and editing. **Elisa Fernandez Fueyo:** Data curation; methodology; project administration; writing – review and editing. **Gemma Budworth:** Data curation. **Dorina Cocirla:** Data curation. **Jennifer Todd:** Data curation; writing – review and editing. **Jane Aspell:** Data curation; writing – review and editing. **Mateo Leganes:** Data curation; writing – review and editing. **Dawn Watling:** Supervision; writing – review and editing. **David Plans:** Software; supervision. **Rebecca Brewer:** Supervision; writing – review and editing. **Jennifer Murphy:** Conceptualization; data curation; formal analysis; funding acquisition; investigation; methodology; project administration; resources; supervision; writing – original draft; writing – review and editing.

## FUNDING INFORMATION

This work was supported by a British Academy/Leverhulme Small Grant to JM (SRG20\200146) and by a Pilot Grant from the Center of Alcohol and Substance Use Studies at Rutgers University. RS is funded by an ESRC South East Network for Social Sciences (SeNSS) PhD studentship (ES/P00072X/1). RB is supported by a Medical Research Council New Investigator Grant (MR/S003509/1). EFF is funded by Royal Holloway, University of London and the Biotechnology and Biological Sciences Research Council (LIDo DTP; grant number BB/T008709/1).

## CONFLICT OF INTEREST STATEMENT

JM has completed paid consultancy work for Healios for work on interoception.

## Supporting information


Data S1.

## Data Availability

The data that support the findings of this study are available from the corresponding author upon reasonable request.

## Footnotes

^1^
Although the terms gender and sex refer to one's identity and biological characteristics, respectively, we use the term sex as almost all interoception‐related research to date has used these terms interchangeably and has focused on individuals whose gender identity aligns with their biological sex.

^2^
This threshold was pre‐registered for Dataset 1.

^3^
Note that removing this individual generally produced a stronger difference where observed.

^4^
While we restricted analyses to 17 valid trials for all analyses, participants completed 20 trials in total. As the amount of valid trials completed across the task may indicate engagement, we included this as a measure of task adherence.

^5^
This threshold was pre‐registered for Dataset 1.

^6^
As this variable was not normally distributed, a nonparametric test was used.
